# Coordinated Changes in Gene Expression Throughout Encystation of *Giardia intestinalis*

**DOI:** 10.1371/journal.pntd.0004571

**Published:** 2016-03-25

**Authors:** Elin Einarsson, Karin Troell, Marc P. Hoeppner, Manfred Grabherr, Ulf Ribacke, Staffan G. Svärd

**Affiliations:** 1 Department of Cell and Molecular Biology, BMC, Uppsala University, Uppsala, Sweden; 2 Department of Microbiology, National Veterinary Institute, Uppsala, Sweden; 3 Department of Medical Biochemistry and Microbiology, Uppsala University, Uppsala, Sweden; 4 Christian-Albrechts-University of Kiel, Institute of Clinical Molecular Biology, Kiel, Germany; University of Zurich, SWITZERLAND

## Abstract

Differentiation into infectious cysts through the process of encystation is crucial for transmission and survival of the intestinal protozoan parasite *Giardia intestinalis*. Hitherto the majority of studies have focused on the early events, leaving late encystation poorly defined. In order to further study encystation, focusing on the later events, we developed a new encystation protocol that generates a higher yield of mature cysts compared to standard methods. Transcriptome changes during the entire differentiation from trophozoites to cysts were thereafter studied using RNA sequencing (RNA-seq). A high level of periodicity was observed for up- and down-regulated genes, both at the level of the entire transcriptome and putative regulators. This suggests the trajectory of differentiation to be coordinated through developmentally linked gene regulatory activities. Our study identifies a core of 13 genes that are consistently up-regulated during initial encystation. Of these, two constitute previously uncharacterized proteins that we were able to localize to a new type of encystation-specific vesicles. Interestingly, the largest transcriptional changes were seen in the late phase of encystation with the majority of the highly up-regulated genes encoding hypothetical proteins. Several of these were epitope-tagged and localized to further characterize these previously unknown genetic components of encystation and possibly excystation. Finally, we also detected a switch of variant specific surface proteins (VSPs) in the late phase of encystation. This occurred at the same time as nuclear division and DNA replication, suggesting a potential link between the processes.

## Author Summary

The intestinal protozoan parasite *Giardia intestinalis* and many other medically important protozoan parasites must encyst and form infective cysts in order to transmit to new hosts. Encystation efficiency is in that way connected to efficiency of transmission. We have developed new *in vitro* differentiation protocols and made the first RNA-seq based gene expression study of the complete *Giardia* encystation process. Our data provides a road map of *Giardia* encystation and a starting point from where it is possible to further explore important processes occurring during encystation. Information about this vital process for survival in the environment of this and other cyst forming parasites can be used in the development of new types of interventions.

## Introduction

*Giardia intestinalis* is an intestinal protozoan parasite of both humans and other mammals and a major cause of diarrheal disease worldwide [1,2]. Its life cycle consists of two stages; the highly motile trophozoite colonizing the small intestine and the dormant cyst excreted in the feces of the host [1,3]. The differentiation process into infectious cysts, known as encystation, is crucial for the parasites transmission and survival. Cysts are usually spread via contaminated water where they can remain infectious for several months if the temperature is low. The infectious dose can be as low as ten cysts [1], which upon ingestion are triggered to excyst, giving rise to four trophozoites per cyst [4]. Many other medically important protozoan parasites are also transmitted as cysts or oocysts (e.g. *Entamoeba histolytica*, *Cryptosporidium parvum/hominis* and *Toxoplasma gondii* [5,6]) but relatively little is known about the differentiation processes due to a lack of *in vitro* systems for encystation. Efficient *in vitro Giardia* encystation and excystation protocols have existed for almost 30 years [7,8]. This makes *Giardia* an excellent model system for studying protozoan cyst formation.

The cyst is characterized by a robust wall that is composed of 40% protein and 60% (ß1-3)-linked GalNAc homopolymer (giardin, [9]), the latter being produced through an inducible synthetic pathway using glucose as a starting substrate [10]. In turn, the protein component is comprised of three major cyst wall proteins (CWP 1–3), which are transported to the cell surface via encystation specific vesicles (ESVs, [11,12]). Encystation can be divided into an early and late phase [13]. In the early phase the adhesive disc and the cytoskeleton is disassembled, the cell starts to round up, CWPs and cyst wall sugars are synthesized and ESVs form. In the late phase nuclei are divided, DNA is replicated and the cyst wall is completed and matured [4].

The structural changes are accompanied by extensive changes in gene expression, a process that was previously investigated using both transcriptomics [14,15] and proteomics [16,17]. Most significantly, these studies revealed that there is a conserved set of genes up-regulated early in encystation [15]. However, a more detailed view on the gradual gene expression changes during the whole encystation process from trophozoite to cyst using the same comparable encystation protocol has been lacking, leaving many aspects of late encystation poorly defined.

In order to increase the robustness and potential to probe this cellular differentiation, we developed a new and improved *Giardia in vitro* differentiation protocol that produces a higher level of mature cysts. We used this new protocol in combination with strand specific RNA-seq to increase the resolution of gene expression changes throughout the entire encystation. Our results reveal that a large part of the parasite transcriptome is altered in a coordinated manner upon the triggering of encystation, with the majority of changes occurring during late encystation. In addition, several novel and previously uncharacterized encystation-specific genes were further characterized.

## Methods

### Cell culture and differentiation

Unless otherwise indicated, reagents were obtained from Sigma Chemical Co. Trophozoites of the *G*. *intestinalis* isolate WB-C6 (ATCC catalog number 50803) were grown axenically in TYI-S33 media supplemented with 10% adult bovine serum (Gibco, Thermo Fisher Scientific) and bovine bile (final concentration 0.125 mg/ml) at pH 6.8 as previously described [18]. Cells were cultivated in T25 culture flasks (Sarstedt) or slanted polystyrene screw cap tubes (Nunc) to 70–80% confluency, when growth media was decanted and pre-warmed encystation media was added to induce encystation. Three different protocols were evaluated for yield of mature cysts using flow cytometry. For the standard 2-step protocol [7], cells were grown in pre-encystation media (pH 7.0) lacking bile for 44 h and thereafter in encystation media (pH 7.8) supplemented with porcine bile (0.25 mg/ml) and lactic acid (5 mM) for 48 hours prior to harvesting. Encystation was also induced by cholesterol starvation using bile-free media (pH 7.85) containing delipidated fetal calf serum [19] in which cells were allowed to differentiate for 24 hours prior harvesting. The third approach, referred to as high bile or the Uppsala encystation protocol, used TYI-S33 supplemented with 10% adult bovine serum and 2.5–5.0 mg/ml bovine bile at pH 7.8. Note that the amount of bile can be varied in order to reach maximum effect depending on the response of the starting cell culture.

Excystation of *in vitro* generated cysts were performed using an improved protocol based on the method from Boucher and Gillin [8]. Pre-warmed induction solution (6.8 ml of 1x Hank’s balanced salt solution containing 32.5 mM reduced Glutathione and 57 mM L-cysteine, mixed with 6.8 ml 0.1M NaHCO_3_ with addition of water to final volume 25 ml) with pH 2.5 was added to the cysts. Cysts were incubated at 37°C using a Thermomixer with shaking at low rpm for 30 min. After removal of induction solution, the cysts were incubated for 40 min in warm excystation solution (15 mg Trypsin and 40 mg Taurocholic acid dissolved in 18 ml 1x Tyrode’s solution, pH 8.0). Excysting cells were transferred to warm growth media to allow proliferation for one hour before numbers of excyzoites/trophozoites and cysts were determined. Excystation frequency was calculated by dividing the number of excyzoites/trophozoites by the number of type 1 cysts [8]. For IFA, excyzoites were fixed in 2% pfa after incubation in excystation solution or after one hour in TYI-S33. Thereafter the cells were added to slides and IFA performed as described below.

### Flow cytometry

Flow cytometry analysis was performed after fixation and staining according to Reiner et al [20]. Approximately 10000 cells per sample were analyzed using a BD LSRII Flow Cytometer at the BioVis Facility (SciLifeLab, Uppsala, Sweden).

### RNA extraction and RNA sequencing

RNA was isolated from non-induced trophozoites and from cells at 1.5, 7 and 22 hours post induction (p.i.) using the Uppsala encystation protocol. At 32 h p.i., cysts were harvested and stored in ddH_2_O at 4°C for three days prior RNA extraction. All samples were extracted using Trizol reagent as described in Franzén et al, 2013 [21]. Purified total RNA was used for library preparation and RNA sequencing at the SNP/SEQ facility of the SciLifeLab National Genomics Infrastructure (Uppsala, Sweden). Generated reads were mapped to the reference genome obtained from GiardiaDB (release 2.4) and the transcriptional analysis was performed as described in Einarsson *et al*, 2015 [22].

### Transcriptome analysis

Each time point during encystation was compared to un-induced trophozoites and a subset of genes with a fold change ≥2 and FPKM values ≥50 in at least one time point were considered significant and used to detect functional clusters within the datasets by the DAVID algorithm version 6.7 [23]. Only groups with enrichment scores ≥1.3 was used for further analysis. In order to visualize temporality of differently expressed genes we used an approach described previously with minor modifications [24]. Briefly all genes with a fold change of ≥2 in any comparison between time points and FPKM values >50 were considered differently expressed (n = 3106). FPKM values were Log_2_ transformed and used in the make.heatmap1 function distributed in the R-project NeatMap package, a package that implements the non-metric MDS algorithm. Smaller heat maps with genes of relevance for transcriptional regulation, glycolysis and variant surface proteins were generated in the same way.

### Real time quantitative PCR analysis

In order to evaluate the accuracy of the RNA sequencing data in respect to gene expression temporality and amplitude, we used real time quantitative PCR (qPCR). We selected 12 differentially expressed genes as targets based on differences in amplitude and expression profiles along the trajectory of the encystation. Primers for the 12 genes displaying differential expression at different times during the encystation and two endogenous control genes were designed and evaluated for PCR efficiency and selectivity (S5 Table). Quadruplicate amplification reactions were performed for each gene and time post-induction of encystation in 10 ml containing balanced amounts of Superscript IV reverse transcribed RNA, SYBR green (Thermo) and 300 nM of both forward and reverse primer. The same was also done on three additional biological replicates of encystation (including 0, 7, 22 and 32 h post induction), resulting in four biological replicates assayed by qPCR. Data was analyzed by computing efficiency corrected relative quantities [25] using the endogenous control gene *tryptophanyl-tRNA-synthetase* as reference (the second control gene *histidyl-tRNA-synthetase* resulted in highly similar quantities) and trophozoite samples as calibrators. qPCR based relative quantities for all time points and genes were thereafter plotted against fold-changes obtained by RNA sequencing and a correlative index was computed by Pearson correlations.

### Vector construction and transfection

Candidate genes for characterization using localization studies were selected based on their highly specific peak expression during encystation. Genes encoding “hypothetical” proteins were prioritized. Genes were amplified with their endogenous promoter regions (for primer sequences see S5 Table) and cloned into the integration vector pPacV-Integ-HA-C [15] or the episomal pPAC-3xHA-C vector [26] using XbaI/PacI and MluI/NotI restriction sites, respectively. Electroporation was performed as described in [26]. Transgenic parasites were selected by the addition of puromycin (50μg/ml) approximately 16 h after transfection and stable transfectants were generally obtained after a week.

### Immunofluorescence analysis

Transfected cells were induced to encyst and harvested at the same time points as for RNA-seq. Cells were washed three times with cold PBS and deposited as monolayers on poly-L-lysine-coated multi-well slides (Thermo Fisher Scientific). Fixation and blocking was carried out as described in [27]. Water resistant cysts were added to the wells of the slide and allowed to air dry prior fixing with 2% PFA. The immunofluorescence assay (IFA) was mainly performed as described in [27]. For HA epitope tagged proteins, the primary antibody used was monoclonal anti-HA rabbit (C29F4, cat.no. 3724,Cell Signaling) diluted 1:1600 followed by incubation with the secondary antibody donkey anti-rabbit Alexa Fluor 594 (1:800) (cat.no.A-21207, ThermoFisher). Alternatively, the HA-tag was detected by monoclonal HA.11 Clone 16B12, Alexa Fluor 594 conjugated (A594-101L, Covance). The monoclonal mouse anti-CWP1 conjugated with Alexa Fluor 488 (Warerborne Inc, New Orleans, LA, USA) was used at dilution 1:100. Fluorescein labeled WGA (FL-1021, Vector Laboratories) diluted to 2 μg/mL was used to visualize ECVs in encysting cells. The slide was air dried and mounted using VectaShield (Vector Laboratories) containing 4’,6-diamidino-2-phenylindole (DAPI) for detection of nuclear DNA. Cells were visualized using a Zeiss Axioplan 2 fluorescence microcope. Images were processed with ZEN grey software (Carl Zeiss GmbH). Super resolution images were acquired for cells encysting 22 hrs p.i labeled with anti-CWP1 antibody (green) to visualize ESVs using a Zeiss LSM710/SIM confocal microscope. Image Z-stacks generated for green and blue (DAPI stain) channels were processed with the ZEN black software (Carl Zeiss GmbH) and images are shown as maximum intensity projections (MIP).

### Western blot

The samples for SDS-PAGE were prepared as described in [26]. Protein samples were separated on SDS-PAGE gels (Any kD, Mini-PROTEAN TGX, Bio-Rad) and transferred to PVDF membranes by electroblotting using standard techniques. The HA tagged proteins were probed using rat anti-HA high affinity clone 3F10 (Roche) diluted to 1:1500 in PBS-T containing 3% BSA for 2h at room temperature. Bound antibodies were detected with horseradish peroxidase conjugated secondary antibody (goat anti-rat HRP, Thermo Scientific) diluted 1:10000 in 3% milk. The blots were developed by using Clarity Western ECL Substrate (Bio-Rad) and images were recorded on a Chemi-Doc MP (Bio-Rad).

## Results

### Evaluation of encystation media

**Fig 1:**
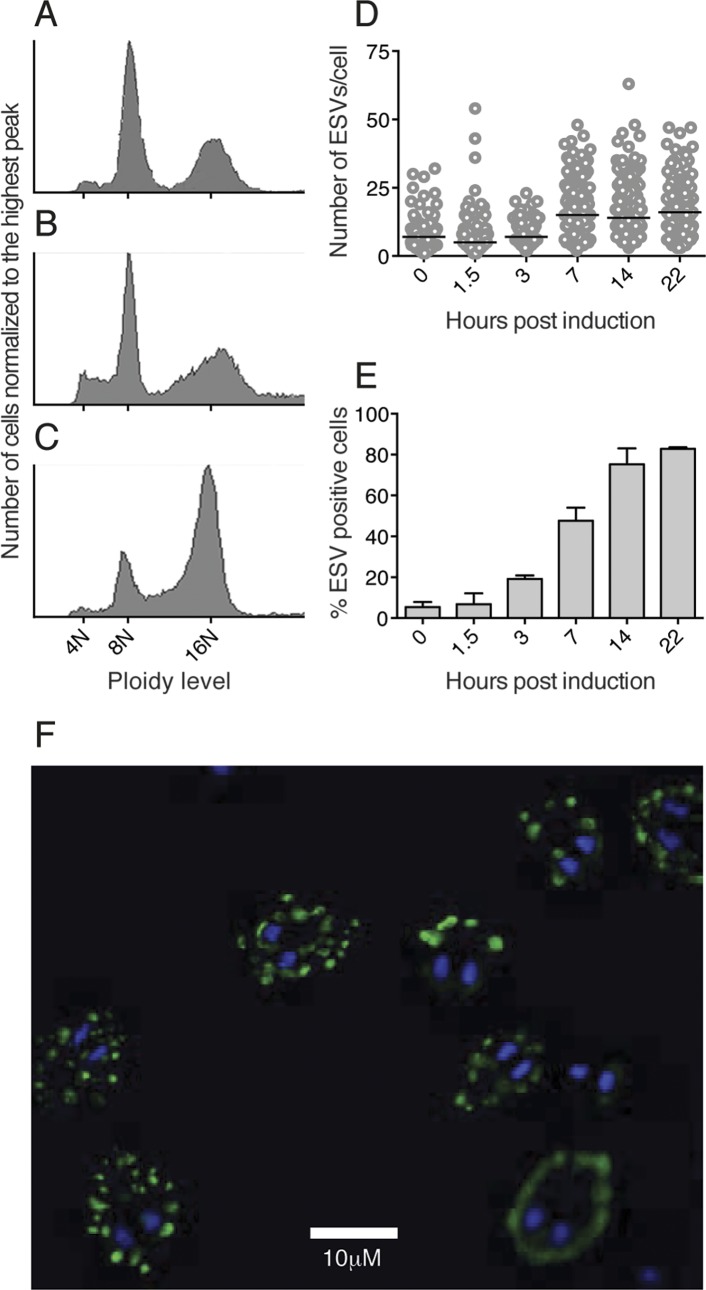
Evaluation of different encystation protocols and ESV formation. (A-C) Flow cytometry analysis of three different encystation protocols in regard to mature water resistant 16N cysts. (A) and (B) histograms represents the cellular distributions in ploidy generated with the standard two-step method and lipid starvation method respectively. The Uppsala encystation protocol (C) generated a higher proportion and yield of mature 16N cysts. Encystation kinetics in the newly developed protocol was evaluated by counting ESVs/cell (D) and the percentage of ESV positive cells (E) within the encysting cell population using monoclonal anti-CWP1 antibody for ESV detection. The data represents three biological replicates with the median of the ESV/cell distribution denoted by horizontal bars (D) and error bars representing the standard deviation (E). (F) ESVs were visualized using super resolution microscopy. Cells were induced to encyst for 22 h and ESVs and nuclear DNA were labeled using an anti-CWP1 antibody (green) and DAPI (blue) respectively. Maximum intensity projections (MIP) of image stacks are shown and the scale bar denotes a distance of 10 μm.

Several protocols have been used to induce *Giardia* encystation *in vitro* and all share the features of lipid starvation and an elevated pH to trigger the process [7,19,28]. In order to increase the resolution of the entire differentiation an additional encystation protocol was developed (Uppsala encystation, see Materials and Methods), aiming to increase the amount of mature 16N cysts and to enable detailed studies of the late phase of encystation as well as mature cysts. Improved yields over the commonly used two-step protocol [7] and the cholesterol starvation method [19] was demonstrated using flow cytometry (Fig 1A–1C).

The encystation kinetics of this protocol was evaluated by counting the number of encystation specific vesicles (ESVs) at five different time points, using an antibody against the encystation marker CWP1 [29] and calculating the percentage of encysting cells in the population. There were large variations in the number of ESVs per cell in the populations (Fig 1D), showing that the differentiation process is asynchronous, as previously observed by others [15,20,30]. However, the median of ESVs/cell and the total amount of encysting cells increased greatly at 7 h post induction (Fig 1D and 1E). After 14 and 22 h the majority (62 and 83%) of the population was encysting (Fig 1E). Super resolution microscopy of 22 h encysting cells showed donut shaped ESVs but also that the ESV morphology and cell shape changes during the encystation process, as described earlier (Fig 1F, [31,32]).

Encystation was allowed to continue for 32 h to obtain maximum amount of cysts. After water treatment for 24 h the numbers of cysts were counted, which revealed a typical yield of 8x10^6^ cysts from a starting culture of 2x10^7^ trophozoites, an encystation efficiency similar to the earlier described protocols [7,19,28]. The cysts obtained by this protocol could also be excysted to a higher extent compared to the cysts produced by the standard two-step protocol (S1 Fig). Thus, this new method for encystation of *Giardia* yields higher levels of mature cysts, making it possible to study the later phases of encystation and also excystation at greater resolution.

### The transcriptional response during encystation

We studied the total gene expression profiles during the complete encystation process (0, 1.5, 7 and 22 h post induction of encystation and water resistant cysts) using the Uppsala encystation protocol and RNA-seq (S1 Table). We used strict selection criteria of FPKM values >50 and fold change >2 for all genes used for further analysis. Genes with different expression profiles and amplitudes were selected for verification (see Materials and Methods) by real time quantitative RT-PCR (qPCR). All of the 12 tested genes displayed a high level of agreement (Pearson r = 0.877) between RNA sequencing data and qPCR data performed on four biological replicates of encystation (S2 Fig). This suggests a high reproducibility of the Uppsala encystation protocol in respect to both timing and level of gene expression during this differentiation process.

**Fig 2:**
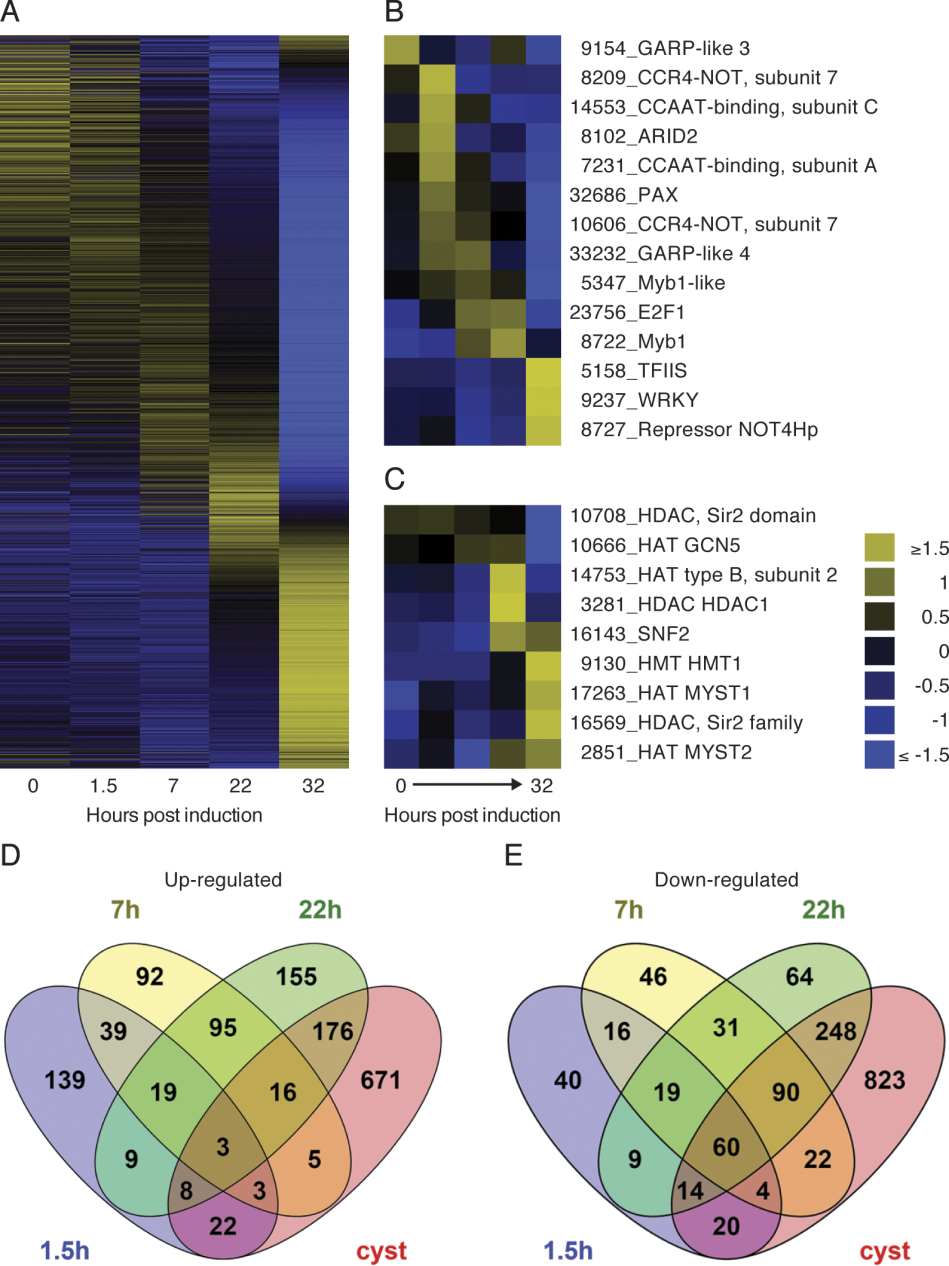
Developmental regulation of the transcriptome along the trajectory of encystation. (A) Non-clustering heatmap of all genes with FPKM ≥50 displaying a fold change ≥2 at any time along the encystation (n = 3106). Yellow and blue indicate up- and down regulated genes respectively on a Log_2_ scale. The cascade fashion of the global transcriptional changes together with similar periodicity for known and putative transcription factors and repressors (B) and chromatin modifiers (C) suggest a coordinated regulation of encystation on both the level of DNA accessibility and transcriptional activation. (D and E) Venn diagrams describing the numbers of unique and shared up- and down-regulated genes for the different time points post induction of cyst formation compared to non-induced trophozoites. The majority of transcriptional changes occur late in the differentiation (22h and cysts) with the majority of genes encoding hypothetical proteins.

We thereafter set out to characterize the periodicity of gene expression along the trajectory of the encystation, by visualizing the expression of all transcripts displaying variable levels in at least one time point in a non-clustered heatmap (Fig 2A). The apparent cascades of up- and down-regulated genes along this trajectory argue for coordinated and developmentally linked gene regulatory activities. This prompted us to investigate the profiles of known and putative mediators of transcriptional regulation in the parasite. Indeed, both known and putative transcription factors and repressors (Fig 2B) and chromatin modifiers such as histone deacetylases (HDACs), histone methyl transferases (HMTs), histone acetyl transferases (HATs) as well as the putative chromatin remodeling complex member SNF2 (Fig 2C) displayed a similar temporality. Collectively this suggests that the entire encystation process is regulated in a coordinated manner on both the level of DNA accessibility and transcriptional activation. This also confirms previous findings that suggest Myb1 as a key mediator in the early phase of encystation [15,33] and that epigenetic mechanisms are at play in the differentiation into transmissible cysts [34].

When the transcriptomes at each encystation time point were compared to the transcriptome in non-encysting trophozoites, it was evident that the major transcriptomal changes occur late in encystation (Fig 2A). Among the genes that were differentially expressed a substantial number of genes encode hypothetical proteins (S1 Table). Venn diagrams were generated to visualize the number of genes with changed expression between time points (Fig 2D and 2E), and revealed that a large number of transcripts are specifically induced or reduced in abundance at discrete stages of *Giardia* encystation (Fig 2D and 2E).

The functional annotation tool DAVID (https://david.ncifcrf.gov/) was used to uncover if any enrichment of function-related gene groups were present at each time point. The functional cluster analysis was performed on both differentially up- and down-regulated genes and only clusters with enrichment scores of ≥1.3 were included (S2 Table). The majority of differentially expressed genes encoding hypothetical proteins were excluded from the cluster analysis due to the inability to assign gene identification numbers. The data from the DAVID analysis is summarized in Table 1 and highlights that certain cellular processes change at specific stages of encystation.

**Table 1:** Functional annotation cluster analysis of differentially expressed genes using DAVID.

Cluster	E-score	No. of genes
**Up-regulated**		
***1*.*5 h***		
ARF/Sar superfamily	1.8	7
EGF-like	1.5	22
***7 h***		
EGF-like	6.0	55
Glycolysis	5.0	13
Ribonucleoprotein complex	4.4	21
Co-factor binding	2.0	11
EGF-like, type 3	1.4	6
***22 h***		
EGF-like	5.8	56
Glycolysis	2.7	11
Co-factor binding	2.2	11
***Cysts***		
DNA binding	3.3	33
Kinase regulator activity	1.7	5
**Down-regulated**		
***1*.*5h***		
Cytoskeleton	1.8	18
Histone core	1.7	12
***7 h***		
Integral to membrane	1.6	6
***22 h***		
Initiation factor	1.8	10
Translation	1.7	33
Nucleobase catabolic activity	1.7	4
***Cysts***		
Translation	12.2	109
Annexin	2.3	21
Protein folding	2.1	26

### Early phase of encystation

During early encystation (1.5 and 7 h post-induction) genes associated with “ARF/Sar superfamily”, “glycolysis”, “ribonucleoprotein complex” and “co-factor binding” were enriched among up-regulated genes (Table 1). The major event in the early phase of encystation is formation of ESVs. The small GTPases Rab1a, Sar1 and Arf1 are involved in vesicle trafficking and have been shown to be essential for proper formation and maturation of ESVs [35]. The expression of several Rabs (Rab 1a, Rab 2a, Rab11 and Rab 32), Sar1 and Sec proteins (Sec-1, Sec13, Sec20) peak after 1.5 h of encystation (S1 Table), reflecting a preparation for the drastic changes in protein transport needed during encystation. The massive production of the cyst wall proteins CWP-1 and -2 was reflected by a 100-fold increase of transcripts between 1.5 and 7 h of encystation (S1 Table). The increased production of secreted proteins at 7 h was also reflected in the up-regulation of most ribosomal proteins, Sec61 and signal recognition particle components (S1 Table).

*Giardia* trophozoites grown *in vitro* use glucose and substrate level phosphorylation to generate energy [36]. The flow through glycolysis increased at 7 h (S1 Table and S3 Fig), suggesting a higher need for energy during the early phase of the encystation process. The enzymes responsible for synthesizing the sugar component of the cyst wall have been shown to be up-regulated during differentiation [10]. We found that this was reflected in our transcriptional data as many of the enzymes involved in carbohydrate metabolism and pathway formation of the GalNAc sugar were up-regulated at 7 h and peaked at 22 h post-induction (S3 Fig).

Among down-regulated genes, clusters such as “Cytoskeleton”, “Histone core” and “Integral to membrane” were enriched (Tables 1 and S2). Alpha- and beta-tubulins, and several genes encoding dyneins were found in the “Cytoskeleton” cluster indicating that several changes in the cytoskeleton occur early in encystation. This is in agreement with earlier studies showing disassembly of the adhesive disc, flagella and a round cell shape [37].

### Consensus of early encystation genes and characterization of hypothetical proteins up-regulated in the early phase of differentiation

**Fig 3:**
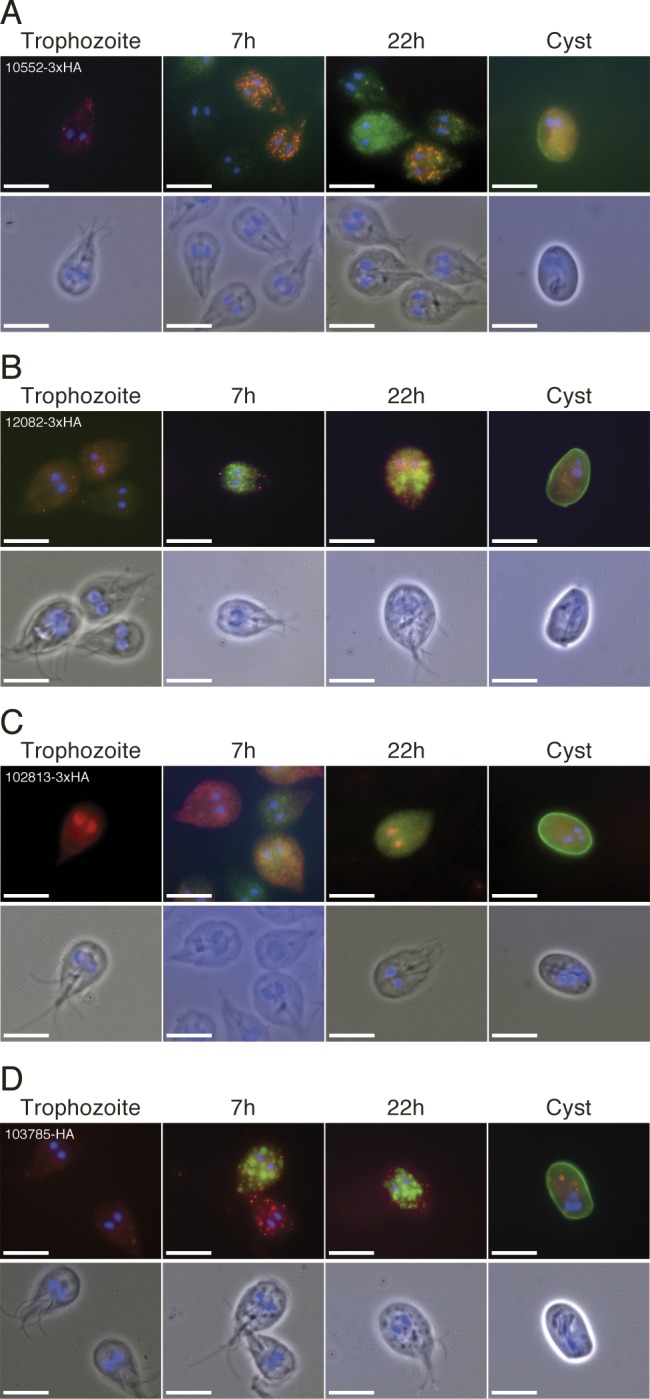
Localizations of epitope tagged proteins of genes in consensus between transcriptional studies. Proteins tagged with HA or 3xHA (red) were visualized in conjunction with CWP1 (green) and DAPI stained DNA (blue) for trophozoites, 7 and 22 h post induction of encystation and cysts. (A) The hypothetical protein 10552-3xHA displayed an ER-like localization in a punctuate manner at both 7 and 22 h. (B) 12082-3xHA appears in vesicle like compartments in early encysting cells and ER like localization in late encysting cells. (C) 102813-3xHA is annotated as a Protein 21.1 and localizes to the nuclei in a proportion of the cells in the population, but not in cysts. (D) 103785-HA localizes to the ER and non-ESV, vesicle-like structures during encystation and in cysts. Scale bars represent 10 μm.

An earlier study of *Giardia* encystation using microarrays and two different encystation protocols generated a core set of 13 genes that are up-regulated early in encystation [15]. We compared our RNA sequencing data with the microarray and SAGE analyses of encystation [14,15] and showed that this set of 13 genes is up-regulated early in encystation in all three studies (S3 Table). The giardial transcription factor Myb1 is part of the consensus list and all 13 genes have Myb binding sites in the promoters [15]. The core group also contains 4 hypothetical proteins (GL50803_32657, 12082, 10552, 3063). Two of the proteins (GL50803_3063 and 32657) have been studied using epitope tagging and were found to localize on the excyzoite surface in the mature cysts [15]. We epitope tagged the two remaining hypothetical proteins (GL50803_10552 and 12082) with HA tags and localized them in encysting cells using CWP1 localization and the nuclei as reference (Fig 3). 10552 localized to a structure reminiscent of the ER in a punctuate manner at both 7 and 22 h post induction of encystation (Fig 3A). 12082 localized to vesicle-like structures in early encysting cells and to a structure reminiscent of ER in late encysting cells (Fig 3B). The protein 21.1 GL50803_102813 is one of the most highly up-regulated genes in the different datasets and it localized to the nuclei in trophozoites and encysting cells but not in the cysts (Fig 3C). We also localized two other of the most highly up-regulated genes early in encystation, hypothetical proteins GL50803_8987 and 103785. They both localized to ER—and vesicle-like structures during encystation (Figs 3D and S4). In the mature cyst they localized to unknown, circular structures (Figs 3D and S4).

### Late phase of encystation

The enriched clusters found at 22 h were genes involved in “Glycolysis” and “Co-factor binding” (Tables 1 and S2). Among the genes associated with glycolysis we detected up-regulation of genes involved in glycogen break-down (S3 Fig). Genes within the “Co-factor binding” cluster included several proteins with pyridoxal phosphate binding specificity. Two of these were serine palmitoyltransferase 1 (GL50803_23015) and serine palmitoyltransferase 2 (GL50803_14374), which are responsible for the first step in the ceramide synthesis pathway. An analysis of all known lipid metabolism genes in *Giardia* [38] showed that there are extensive changes in the lipid metabolism in the late phase of encystation (S1 Table).

The DAVID cluster analysis for down-regulated genes in late encystation revealed enrichment of genes involved in processes such as “Initiation factor”, “Nucleobase catabolic activity” and “Translation” (Tables 1 and S2). The gradual decrease of translation-associated processes was expected as the cells at this point prepare to enter dormancy and protein production arrests.

For the mature cysts, all core histones were up-regulated together with several kinesins and genes associated with meiosis (S1 Table). Many of these genes had similar expression profiles in the SAGE dataset and stayed highly abundant throughout excystation [14]. The up-regulation of histone transcripts could be the result of condensation of the chromatin in the cysts or alternatively that the transcripts were produced to enable the rapid cellular division that the cells undergo shortly after excystation [4]. This could also be the case for the seven kinesins found to be up-regulated, since most of these motor proteins are associated with flagellar assembly, a process vital for the excyzoite as the flagella have been seen to emerge first from the cyst [39]. *Giardia* has several homologs to meiotic genes despite the apparent lack of a sexual cycle [40]. Most of the meiotic genes are up-regulated in the late phase of encystation and in mature cysts (S5 Fig). Three genes with predicted transcription regulatory function were also found up-regulated late in encystation (GL50803_11383, 9237 and 16143, S1 Table). However, we currently do not know their binding specificity and what role they might play in encystation.

Genes associated with processes such as “Translation” and “Protein folding” were down-regulated in mature cysts (Table 1). Calcium-binding proteins, the majority of them alpha-giardins, were down-regulated indicating large cytoskeleton changes in mature cysts. The alpha-giardins are a family of annexin-like proteins found associated with the cytoskeleton and many of these localize to the plasma membrane and/or the flagella [41]. Disc associated alpha-giardins (-2, -3, -5 and -17) were all down-regulated gradually during encystation (S1 Table), as were the disc proteins SALP-1, beta-giardin, gamma-giardin and delta-giardin, reflecting the disassembly of the adhesive disc [37].

### Expressional changes of cysteine-rich proteins during in the late phase of encystation

**Fig 4:**
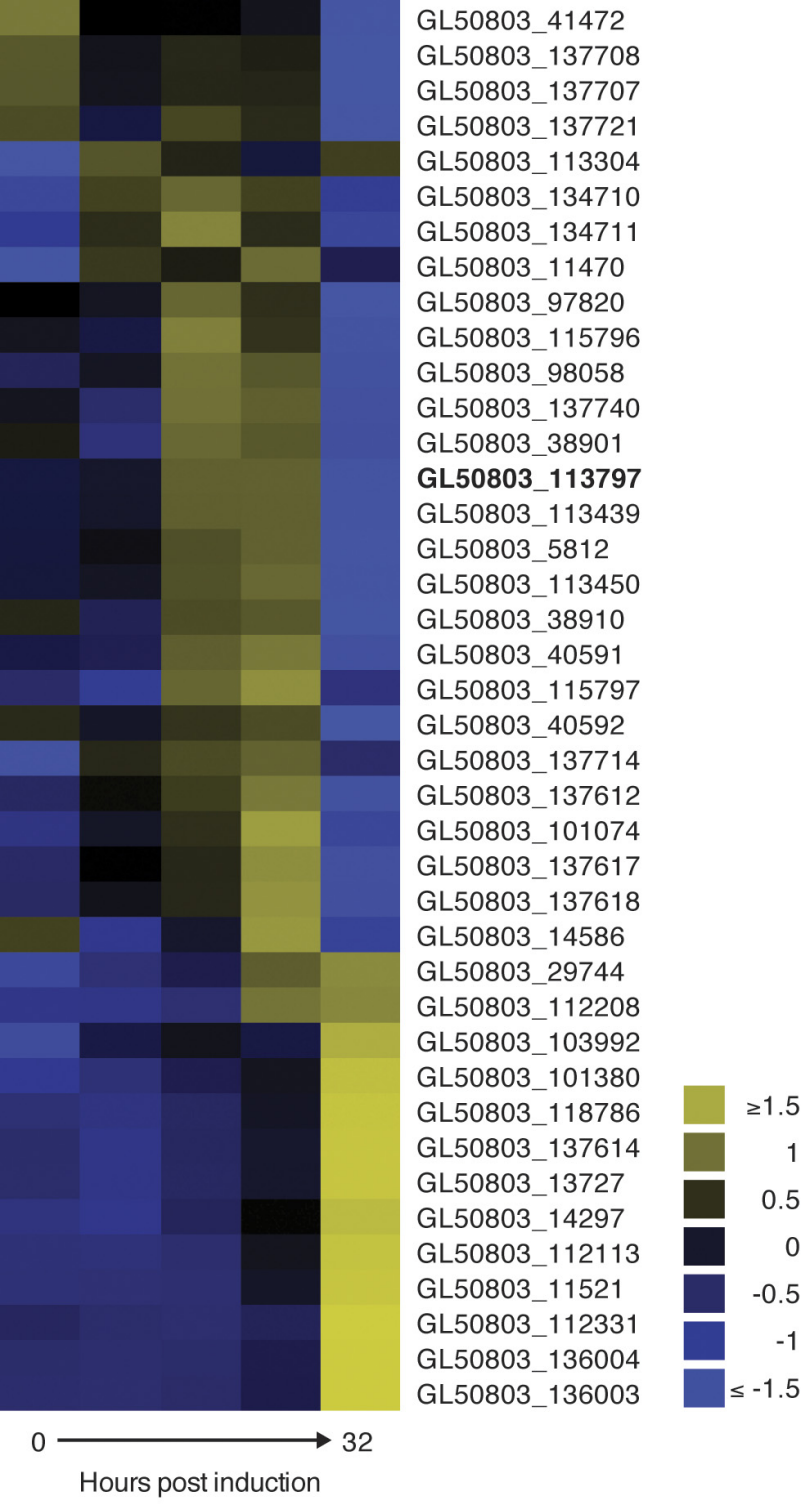
Transcription of VSPs switch during differentiation. Non-clustering heatmap of the highest transcribed VSPs during differentiation reveal changes in VSP expression along the trajectory of encystation. Elevations in abundance of several VSP transcripts not expressed in the starting population are observed at the different time points. The VSP with the highest cumulative level of expression in the population throughout encystation was GL50803_113797 (bolded) also known as TSA 417.

Based on the output of the functional cluster analysis it was evident that expressional changes occurred for genes encoding cysteine rich proteins with EGF domains (Tables 1 and S2). The majority were variant-specific surface proteins (VSPs), cysteine-rich surface proteins involved in antigenic variation [42]. Our transcriptional data suggest that there were expressional changes of several VSPs during encystation (Fig 4). The VSP with highest expression in the starting trophozoite population and in encysting cells was VSP-5 (GL50803_113797, also known as TSA 417, [43]) but it was down-regulated after 22h (Fig 4). The VSPs with highest expression in cysts are not the same as the VSPs expressed in trophozoites (S1 Table and Fig 4). An analysis of the expression-profiles of specific VSPs shows that there were a major up-regulation of VSPs between 22 and 32 h of encystation (S1 Table and Fig 4).

The expression of the VSP-related but less studied High-Cysteine Rich Membrane proteins (HCMp), High-Cysteine Proteins (HCP) [44] and Tenascins [45] also changed during encystation. The high cysteine non-variant surface protein (HCNCp) is the only HCMp that has been characterized so far and it is transported in ESVs to the excyzoite surface during encystation [44]. S4 Table shows that there are extensive changes in expression in all of the 60 HCMp genes during encystation, which was also seen in the 25 HCPs that are similar to HCMPs but with fewer cysteine-rich motifs (S4 Table). A few members of the Tenascin family (10 members in the WB strain) have been studied and they localized to the ESVs and the cyst wall [45]. We detected large gene expression changes in the Tenascin family in the early phase of encystation (S4 Table).

To summarize, there are extensive changes in the VSP-related gene families during the late phase of *Giardia* encystation. Changes in the VSP expression is related to antigenic variation but further studies are needed to elucidate the functions of HCMps, HCPs and Tenascins in the cell and what role they play in the life cycle of the parasite.

### Characterization of hypothetical proteins up-regulated in the late phase of encystation

**Fig 5:**
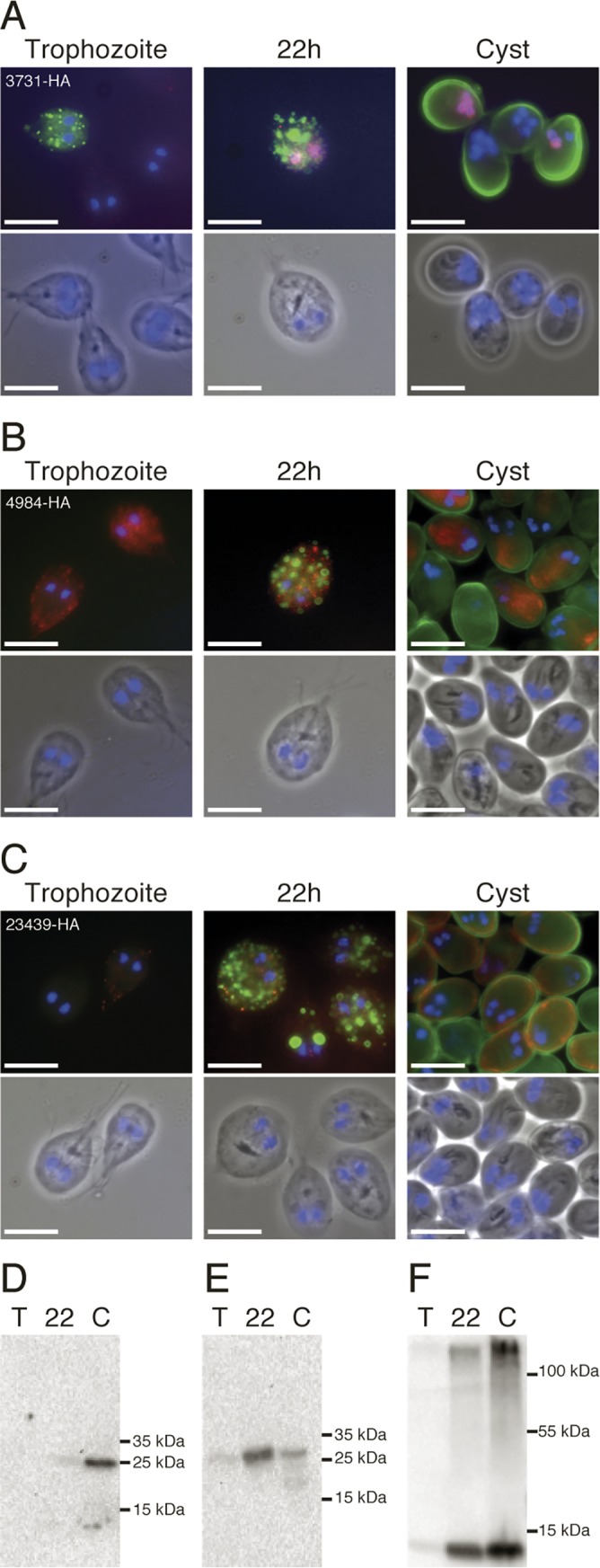
Expression and localizations of epitope tagged proteins up-regulated in the late phase of encystation. Proteins of genes with a transcriptional profile of late induction during encystation were epitope tagged with HA (red) and localized with CWP1 (green) and DAPI stained DNA (blue) by immunofluorescence. Scale bars represent 10μm. (A) 3731-HA localizes to the nuclei of a proportion of encysting cells and cysts. (B) 4984-HA showed localization to an unknown structure in mature cysts and appeared vesicle-like in encysting cells. (C) 23439-HA localized to vesicle-like structures in encysting cells and close to the membrane of the excyzoite in cysts. (D-F) Analysis of corresponding protein levels in extracts from trophozoites, 22h post induction and cysts by western blots reveal a marked increase in the 22.8 kDA 3731-HA (D), 23.6 kDa 4984-HA (E) and 10.8 kDa 23439-HA (F) over time. The larger band for 23439-HA indicates a potential association of the protein to the cyst wall.

The largest group of up-regulated genes in the late phase of encystation comprised hypothetical genes (Fig 2 and S1 Table). In order to begin characterization of a few of these, we epitope tagged 10 different hypothetical proteins with HA-tags and studied their expression and localization. Western blot analysis of hypothetical proteins 50803_3731, 4984 and 23439 verified that they are all up-regulated both at RNA and protein level late in encystation (Fig 5). The HA-tagged proteins were visualized in immunofluorescence using an anti-HA antibody and ESVs and cyst wall were shown using a CWP1 antibody (Fig 5). The three hypothetical proteins do not localize to ESVs during encystation (Fig 5). Protein 3731 localizes to the nuclei at 22 h encystation in a proportion of encysting cells and mature cysts (Fig 5A). Protein 4984 localizes to small, non-ESV puncta at 22h and the excyzoite surface in mature cysts (Fig 5B). Protein 23439 also localizes to non-ESV puncta at 22h and to the excyzoite surface close to the cyst wall (Fig 5C). The presence of a high molecular weight band in the cyst lane of the Western blot (Fig 5F) is similar to what can be seen during Western blot analyses of CWPs [46]. Cysts containing the HA-tagged 23439 protein were excysted, using a new more efficient protocol (Materials and Methods), and the protein localized to the cortex of the excyzoite (S6 Fig). We selected seven additional hypothetical proteins with the same expression profiles to be studied as epitope tagged proteins (S4 Fig). Proteins 4764 and 5062 localized to the nuclei of encysting and mature cysts in a similar pattern as 3731 (S4 Fig). 15594 and 14690 both localizes to an unknown structure in mature cysts and to non-ESV puncta in encysting cells (S4 Fig). 6227, 7374 and 11363 localizes close to the membrane of the excyzoite in mature cysts and 7374 and 11363 show the same high molecular bands as 23439 in the Western blots (S4 Fig).

To summarize, we have verified the up-regulation of 10 hypothetical proteins in the late phase of encystation. The proteins localize to the nuclei, excyzoite surface and to unknown vesicles and cellular structures in encysting cells and cysts.

## Discussion

**Fig 6:**
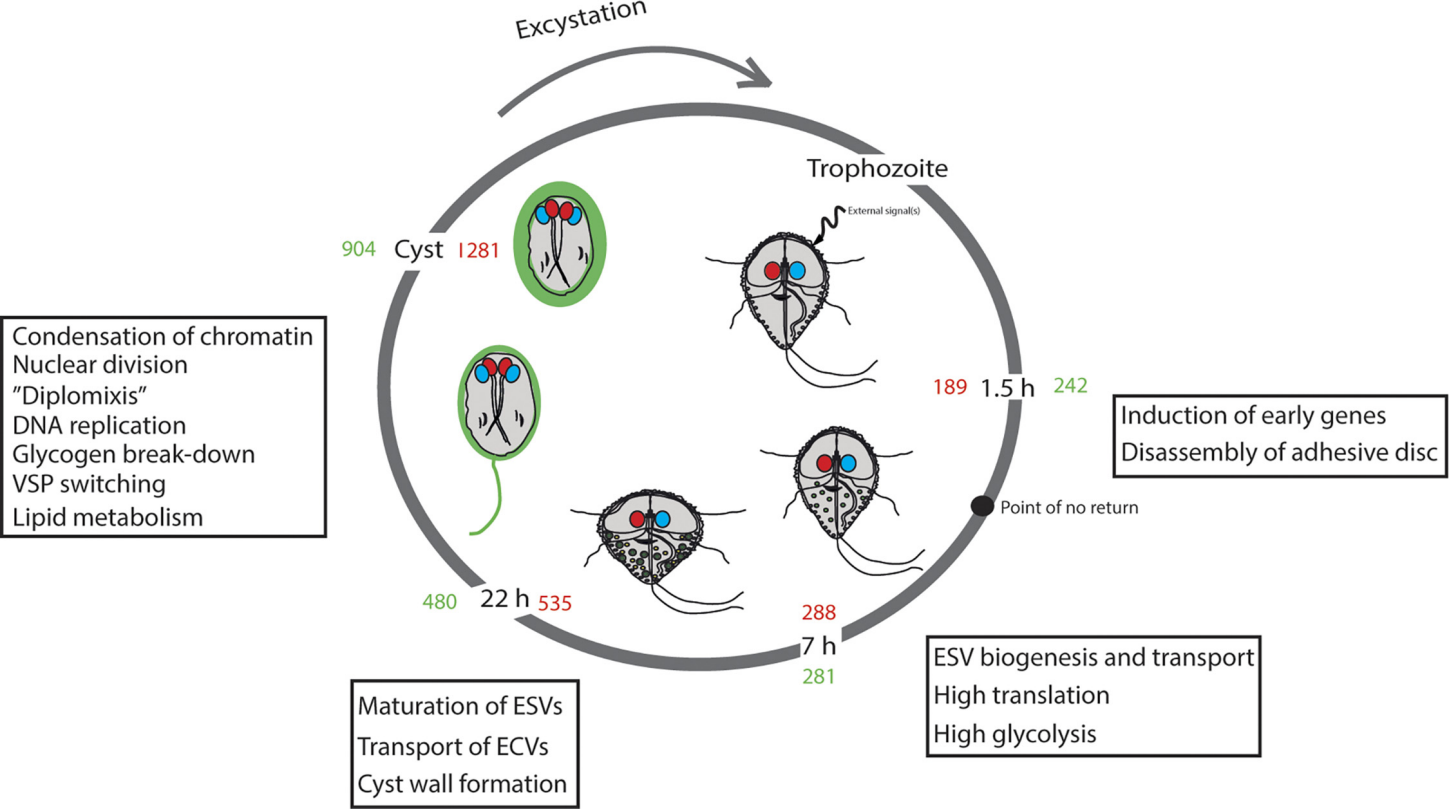
Summarizing image showing the transformation from the motile trophozoite via encyzoite to the final cyst stage. The trophozoite monitors the external environment and encystation is induced via intracellular pathways that remain largely unknown. The cell passes a “point of no return” during early encystation after which it is no longer possible to relapse to the proliferating stage. Transcription factors (e.g. Myb2) activates encystation-specific genes among them are cyst wall proteins (CWP1-3). An overall increase of translation could be observed early in encystation as the production of CWPs is dramatically increased and the transportation in encystation vesicles (ESVs) begins. The vesicles undergo maturation steps after leaving the ER. The other component of the cyst wall, the UDP-GalNAc sugar (giardin), is also synthesized and secreted via encystation positive carbohydrate vesicles (ECVs). The enzymes involved in giardin synthesis are induced during encystation. During late encystation, the cell changes shape as it enters dormancy and the ventral disc together with the flagella are disassembled as the construction of the cyst wall proceeds. But the mechanism behind the assembly is still unknown. Often pre-cyst stages with a “tail” can be observed in encystation. Two rounds of DNA replication occur without cytokinesis rendering a cyst with four nuclei each with the genome ploidy of 4N. Interconnections between the nuclei in the cysts are formed and genetic material can be exchanged through the process “diplomixis”. During excystation, each cell receives one pair of non-sister nuclei (indicated as red and blue).

*Giardia* must differentiate into infective cysts in order to transmit to a new host and therefore encystation is connected to the efficiency of transmission and virulence. We have used a new encystation protocol in combination with RNA-seq in an attempt to generate new information of the encystation process but also to link earlier observations along the encystation time axis. Our results and previously published data are summarized in Fig 6. Taken together, these findings show that the encystation process can be divided into an early and late phase and that distinct processes occur step-wise during encystation.

Cholesterol starvation in the lower part of the small intestine complemented by a raise in external pH has been suggested to be the major signal for induction of *Giardia* encystation [19]. There is very limited knowledge about how intracellular signaling is induced and mediated during encystation. Encystation of synchronized parasites suggests that the parasites leave the cell cycle in the G2 phase of the cell cycle to start encystation [20]. The parasite reaches a “point of no return” early in encystation (3–4 h, Fig 6) after which it is impossible to revert back to proliferation and differentiation will continue until mature cysts are generated [47]. The transcriptome changed after 1.5 h of encystation and 7 h post-induction an even higher number of genes changed their expression (Fig 2). A previous study used microarrays to study the early phase of encystation and revealed a core set of 13 genes consistently induced at 7 h [15], which we could verify (S3 Table). These up-regulated genes all contain at least one Myb binding site, a transcription factor known to regulate encystation-specific genes [33], supporting the earlier observation that the Myb binding site is a signature motif for early phase encystation genes [15].

The most striking change in secretion of proteins occurs during the early phase of encystation when massive amounts of cyst wall components are transported from the ER to the plasma membrane. At 7 h of encystation ribosomal proteins and translation factors peak in expression reflecting the high production of cyst-wall proteins (S1 Table). The cyst wall material (CWM) is transported in ESVs and the formation and transport of these have been extensively studied [11,12]. The cargo of ESVs is exclusively CWM and no other secreted proteins, strongly suggesting sorting in the ER prior to export [11,31,48]. *Giardia* lacks a constitutively expressed Golgi apparatus and the features of ESVs indicate that these organelles act as a stage-induced *cis* Golgi [35]. The trafficking process of CWM, from the ER to the ESV, appears to be dependent on Rab1a, COPII coat formation and the small GTPase Sar1 [35]. In line with this we could see a peak of expression of these genes and other proteins involved in protein transport already after 1.5 h of encystation (S1 Table). The CWPs are not directly secreted after completion of ESV formation but instead delayed for several hours and converted into a trans-Golgi like tubular vesicular network during the late phase of encystation [31]. This is presumably to allow post-translational modifications and complex formation of CWPs prior to secretion at the cell surface. [31].

In contrast to the well-characterized transportation of CWM via ESVs, very little is known about the transportation and incorporation of the GalNAc sugar to the cyst wall. This *Giardia*-unique carbohydrate is synthesized *de novo* by a pathway containing five enzymes that peak in expression at 22 h encystation (S3 Fig) as the flow through glycolysis decreases. The GalNAc homopolymer has been visualized using the GalNAc binding properties of recombinant CWP1 and reported to be present in small vesicles of encysting *Giardia*. These vesicles did not co-localize with ESVs, suggesting a different transportation pathway of the sugar components of the cyst wall and they were named encystation carbohydrate-positive vesicles (ECVs) [49]. ECVs are more prevalent in the late phase of encystation. Further studies are needed to clarify how the ESVs and ECVs are connected and to identify the mechanism behind cyst wall assembly. Interestingly, we identified encystation-induced, non-ESV puncta that contained the hypothetical proteins 23439, 11363, 7373 and 6227 (Figs 5 and S4) and appear to associate to the cyst wall. Additionally, the proteins 4984, 14690 and 15594 appeared in vesicle-like structures during encystation (Figs 5 and S4). ECVs are labeled by the lectin WGA [49] but the proteins 6227, 7374, 11363 and 23439 do not co-localize with WGA (S7 Fig). It remains to be seen if these hypothetical proteins localize to yet another class of encystation-specific vesicles induced late in encystation.

Large overall gene expression changes were identified in the late phase of encystation in an earlier study of the encystation transcriptome using SAGE technology [14]. However, two different encystation protocols were combined in this study (trophozoites to 42 hours encystation via the two-step method and cysts via the high bile method), which made it difficult to draw certain conclusions. Here we used the Uppsala encystation protocol and RNA-seq and we could verify that there are large changes in the transcriptome in the late phase of encystation (Fig 2). It will be important to expand the proteomic studies of encystation and connect it to the identified transcriptional changes during *Giardia* encystation.

During encystation the two nuclei divide without cytokinesis forming a pre-cyst with ploidy of 4x2N followed by another round of replication to the final ploidy of 16N (4x4N) in the mature cyst [4]. Evidence for fusion of the nuclei and exchange of genetic material during encystation has been described [30]. The authors named this event “diplomixis” and three meiotic genes were reported to be expressed late in encystation to facilitate homologous recombination [30]. DNA replication occurs around 15–20 h of encystation using our new protocol. At the same time as DNA is replicated there are extensive changes in gene expression of several genes involved in DNA repair and chromatin modifications (S1 Table). Few transcription factors have been found in the *Giardia* genome [50,51] and earlier data showed that epigenetic changes are involved in the encystation process [34]. We searched for conserved sequences in the up-stream regions of late encystation genes with the same expression profiles, but we only detected the earlier characterized Myb boxes in the giardin synthesis genes [33,52] and the conserved sequences in the histone promoter regions [53]. This is in contrast to the conserved core genes early in encystation with Myb binding sites [15], suggesting that the regulation of gene expression during encystation occurs at multiple levels. It should be noted that there is a need for further RNA-seq studies using additional time-points and biological replicates in order to increase the resolution of the changes in the transcriptome during encystation. This can in turn reveal conserved promoter elements or chromatin modifications in genes with similar expression profiles.

We detected major changes in the expression of variable-specific surface proteins (VSPs) during encystation. VSPs are cysteine rich membrane proteins responsible for antigenic variation. They have variable size (30–150 kDa) and different *Giardia* isolates have between 150–300 VSP genes spread over the 5 chromosomes [54]. Only one VSP is expressed on the protein level per cell and the regulation has been suggested to be post-transcriptional [55,56]. VSP expression changes already early during encystation but in the late phase, (22 h post-induction and cysts) there are large changes in the expression of VSP genes (Fig 4). The dominant VSP expressed in the trophozoite population (GL50803_113797, VSP5, S1 Table) is down-regulated whereas five VSP genes are highly up-regulated (Fig 4 and S4 Table). The first study of antigenic variation in encysting *Giardia* WB parasites [57] found that the same VSP (GL50803_137614, VSP180) as in our experiment dominated in cysts (S4 Table). This shows that certain VSPs are specifically regulated by encystation signals and a switch of VSP expression is initiated around the same time as the DNA is replicated in the late stages of differentiation. Further studies of this encystation-specific switching event can potentially reveal how the *Giardia* parasite can express one or a limited number of VSP genes out of the 200 present in the genome.

## Supporting Information

S1 Table The total transcriptome analyzed by RNA sequencing during encystation of *Giardia intestinalis* (strain WB) where transcription levels are presented as FPKM values for each time post induction of encystation. (XLS)

S2 Table Functional annotation cluster analysis performed using DAVID for genes with variable transcript levels (≥2 fold) at each time point. (XLSX)

S3 Table Consensus table of up-regulated genes at 7h post induction in different encystation protocols assayed by RNA seq, microarrays and SAGE reveal a small consensus set of 13 genes found across all techniques and protocols used. (XLSX)

S4 Table Transcriptional changes of VSPs, High Cysteine proteins (HCMPs and HCPs) and Tenascins during differentiation. (XLSX)

S5 Table List of primers used for cloning and verification by qPCR. (XLSX)

S1 Fig Excystation frequencies of cysts generated by different encystation protocols *in vitro*. Excystation frequencies were compared between cysts generated by the commonly used 2-step method and the newly developed Uppsala encystation protocol. Encystations and excystations of cysts from the two protocols were performed in parallel at three separate occasions (Rep #1–3). Data is displayed as mean excystation frequencies and error bars represent the variation between fields counted. Cysts obtained from the Uppsala protocol excysted at a higher efficiency at all occasions tested.
(TIF)

S2 Fig qPCR verification of RNA sequencing data and robustness of encystation protocol. (A-L) Relative expression profiles of 12 genes showing differential transcript levels along the trajectory of encystation. qPCR based relative quantities at different times post induction of encystation relative to trophozoites were computed using the endogenous control gene *tryptophanyl-tRNA-synthetase* and plotted together with fold-changes observed by RNA sequencing. The RNA sequencing data is represented by squares joined by dashed lines and the qPCR data is shown as circles joined by solid lines. The latter represents average relative quantities from 4 replicate encystations (each assayed in quadruplicates) with error bars representing the ranges of the bio replicates. (M) The high agreement of the two experimental approaches and repeating encystations is reflected in a high correlative index by Pearson (r = 0.877) and suggest the newly developed protocol to be robust and the global transcriptional analysis to be accurate.
(TIF)

S3 Fig Changes in glycolysis during encystation. (A) Non-ordered heatmap of transcript levels from glycolytic genes (Log_2_ scale) reveals a high level of periodicity and encystation stage differences in the catabolism of glucose. (B) Schematic pathway of the *G*. *intestinalis* glycolysis with peak expression levels observed during the encystation marked for individual genes.
(TIF)

S4 Fig Localization of hypothetical genes up-regulated during encystation. Eight HA-epitope tagged proteins were visualized using antibodies to anti-HA (red), CWP1 (green) and DAPI staining of nuclear DNA (blue). All scale bars represent 10µm. (A) The early induced 8987-3xHA localizes to vesicle-like structures in encysting cells and cysts. (B) 14690-3xHA localizes to an unknown structure in mature cysts and to vesicle-like structures in encysting cells. Western blot reveals a protein of the expected size (35.9 kDa) highly increased in cysts. 7374-HA (C), 11363-HA (D) and 6227-HA (E) localize to the membrane of the excyzoite in mature cysts with western blots confirming the late induction and high molecular weight bands indicating association to the cyst wall for 7374-HA (C) and 11363-HA (D). 4764-HA (F) and 5062-HA (G) localize to the nuclei of encysting and mature cysts (F) whereas 15594-3xHA (H) localizes to an unknown structure in mature cysts and to vesicle-like structures in encysting cells.
(TIF)

S5 Fig Transcriptional profile of meiosis related genes. The meiosis related genes found in *Giardia* reveal differential expression during encystation with a marked up-regulation during later stages.
(TIF)

S6 Fig Excystation of 23439-HA transfectant shows protein on surface of excyzoite. Water treated cysts expressing 23439-HA were excysted as described in Materials and Methods. Emerging excyzoites were fixed in solution and the HA-tagged protein was localized using an anti-HA antibody (red) in conjunction with an antibody to CWP1 (green) and DAPI for nuclear DNA staining. The tagged proteins appear on the surface of emerging excyzoites. Scale bars 10 µm.
(TIF)

S7 Fig Localization of WGA and genes up-regulated late in encystation. The proteins 6227, 7374, 11363 and 23439 display localizations in vesicle-like puncta and in order to investigate potential co-localization to ECVs, we used fluorescein labeled WGA. The HA-tagged strains of these proteins were subjected to encystation followed by immunofluorescence analysis to detect potential co-localizations. The WGA labeling (green) displayed ER-like staining and did not co-localize with any of the HA-tagged (red) proteins in encysting parasites or in cysts. Scale bars represent 10 μm.
(TIF)
