# Supplementary figures and images for: Coordinated Changes in Gene Expression Throughout Encystation of Giardia intestinalis
Source: PLoS Negl Trop Dis. 2016 Mar 25;10(3):e0004571. doi: 10.1371/journal.pntd.0004571 (PMC4807828; doi:10.1371/journal.pntd.0004571)

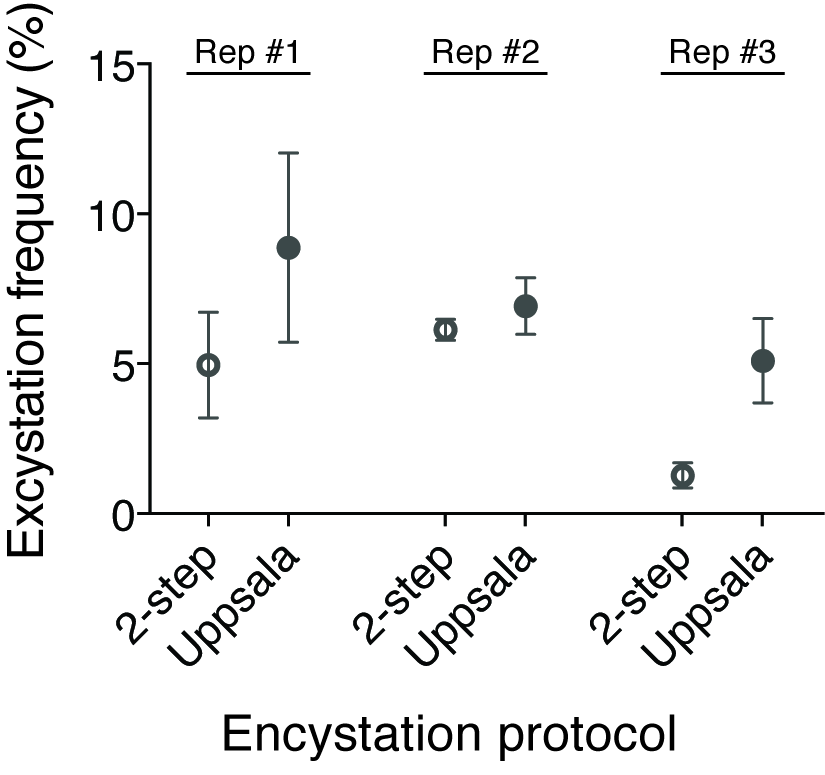

Supplement: S1 Fig — Excystation frequencies were compared between cysts generated by the commonly used 2-step method and the newly developed Uppsala encystation protocol. Encystations and excystations of cysts from the two protocols were performed in parallel at three separate occasions (Rep #1–3). Data is displayed as mean excystation frequencies and error bars represent the variation between fields counted. Cysts obtained from the Uppsala protocol excysted at a higher efficiency at all occasions tested. (TIF) [file pntd.0004571.s006.tif]

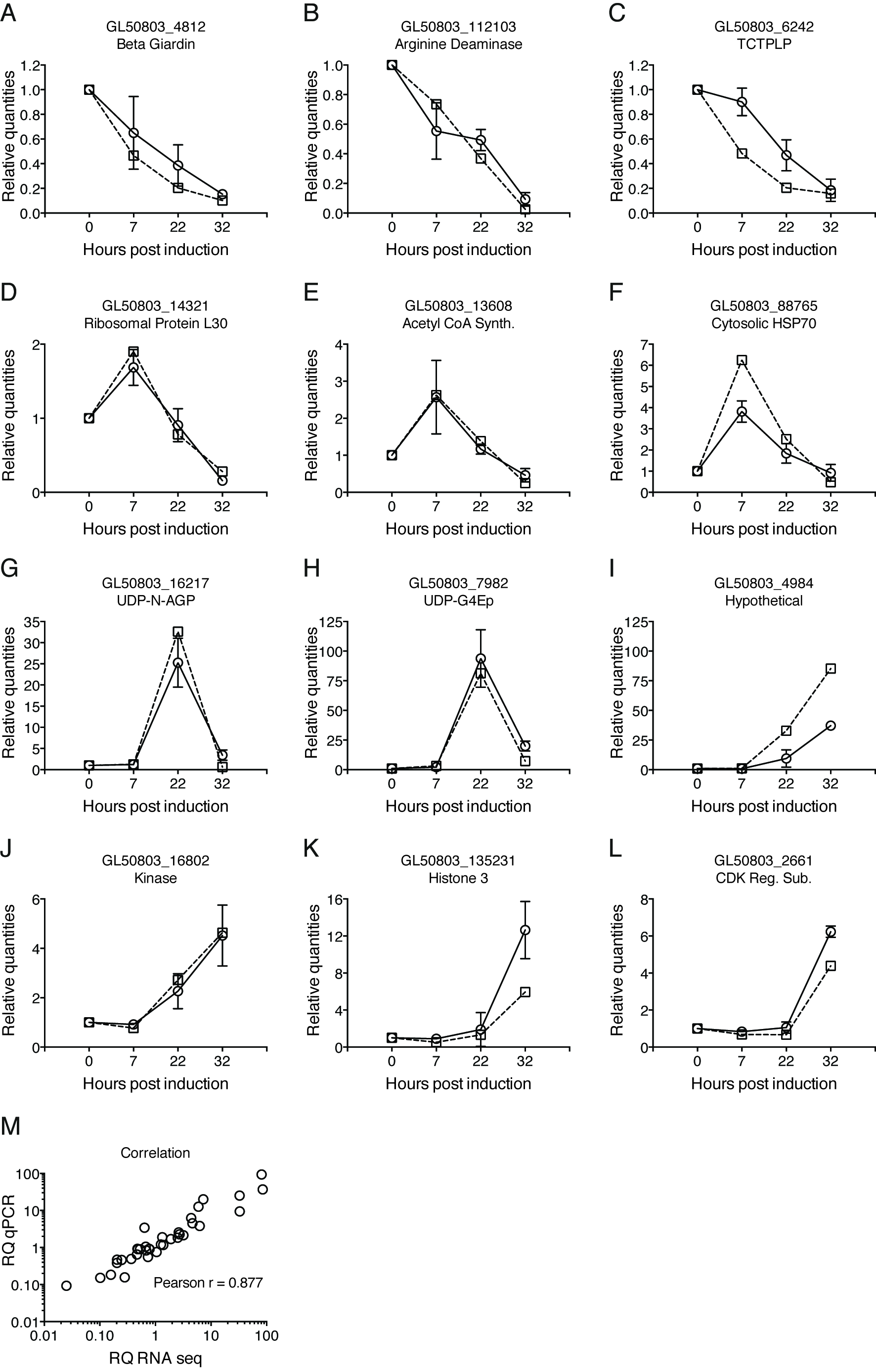

Supplement: S2 Fig — (A-L) Relative expression profiles of 12 genes showing differential transcript levels along the trajectory of encystation. qPCR based relative quantities at different times post induction of encystation relative to trophozoites were computed using the endogenous control gene tryptophanyl-tRNA-synthetase and plotted together with fold-changes observed by RNA sequencing. The RNA sequencing data is represented by squares joined by dashed lines and the qPCR data is shown as circles joined by solid lines. The latter represents average relative quantities from 4 replicate encystations (each assayed in quadruplicates) with error bars representing the ranges of the bio replicates. (M) The high agreement of the two experimental approaches and repeating encystations is reflected in a high correlative index by Pearson (r = 0.877) and suggest the newly developed protocol to be robust and the global transcriptional analysis to be accurate. (TIF) [file pntd.0004571.s007.tif]

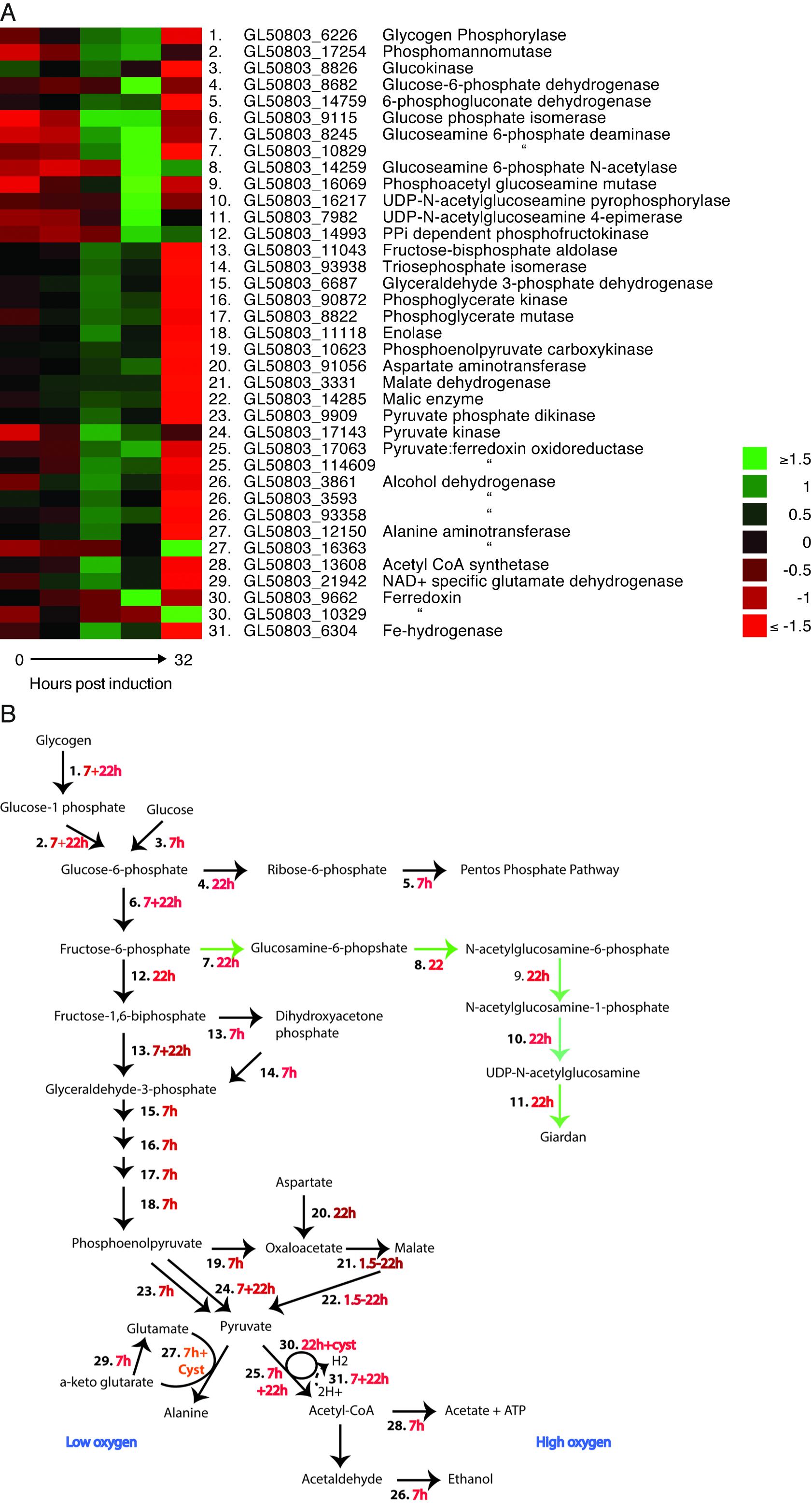

Supplement: S3 Fig — (A) Non-ordered heatmap of transcript levels from glycolytic genes (Log2 scale) reveals a high level of periodicity and encystation stage differences in the catabolism of glucose. (B) Schematic pathway of the G. intestinalis glycolysis with peak expression levels observed during the encystation marked for individual genes. (TIF) [file pntd.0004571.s008.tif]

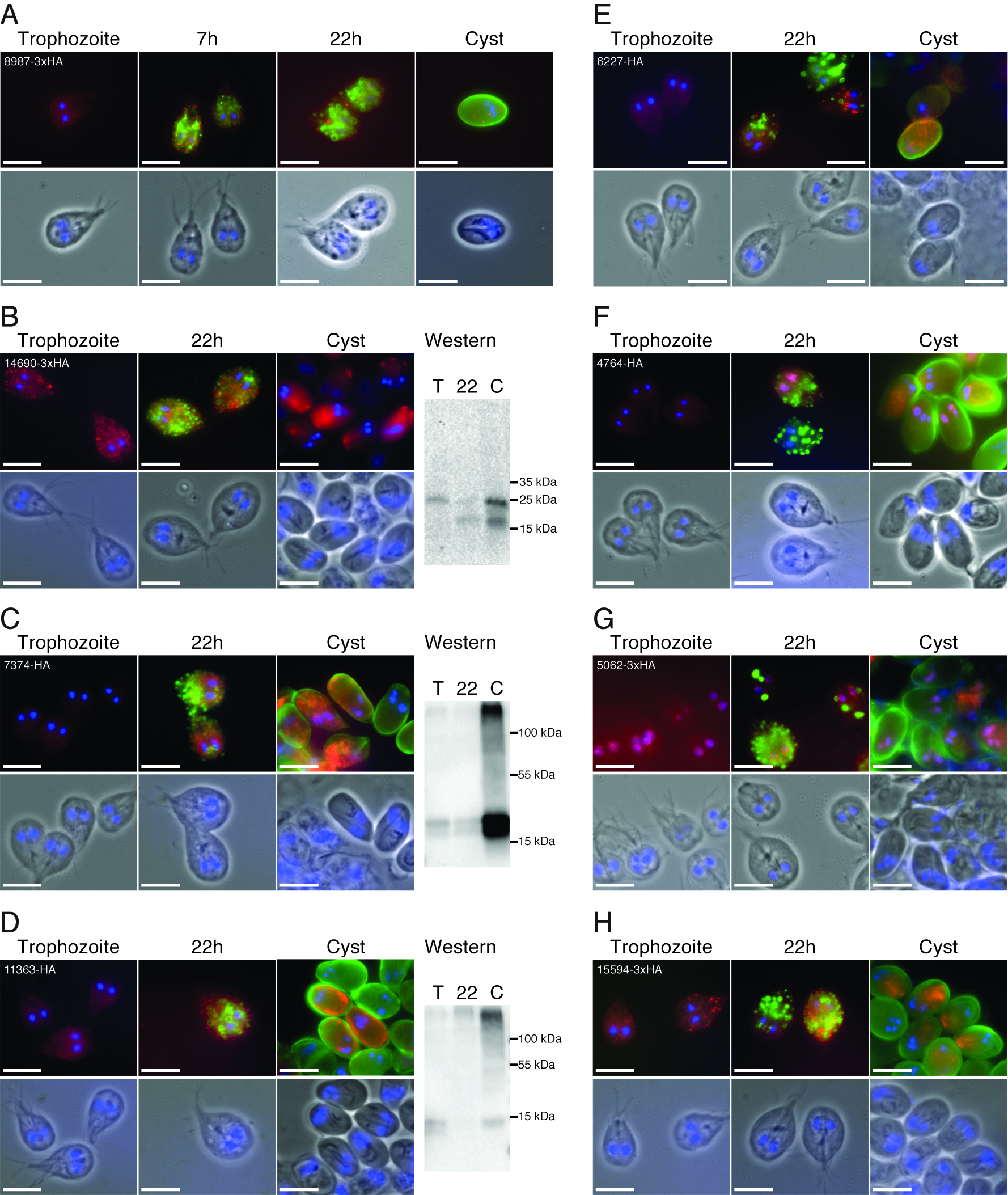

Supplement: S4 Fig — Eight HA-epitope tagged proteins were visualized using antibodies to anti-HA (red), CWP1 (green) and DAPI staining of nuclear DNA (blue). All scale bars represent 10µm. (A) The early induced 8987-3xHA localizes to vesicle-like structures in encysting cells and cysts. (B) 14690-3xHA localizes to an unknown structure in mature cysts and to vesicle-like structures in encysting cells. Western blot reveals a protein of the expected size (35.9 kDa) highly increased in cysts. 7374-HA (C), 11363-HA (D) and 6227-HA (E) localize to the membrane of the excyzoite in mature cysts with western blots confirming the late induction and high molecular weight bands indicating association to the cyst wall for 7374-HA (C) and 11363-HA (D). 4764-HA (F) and 5062-HA (G) localize to the nuclei of encysting and mature cysts (F) whereas 15594-3xHA (H) localizes to an unknown structure in mature cysts and to vesicle-like structures in encysting cells. (TIF) [file pntd.0004571.s009.tif]

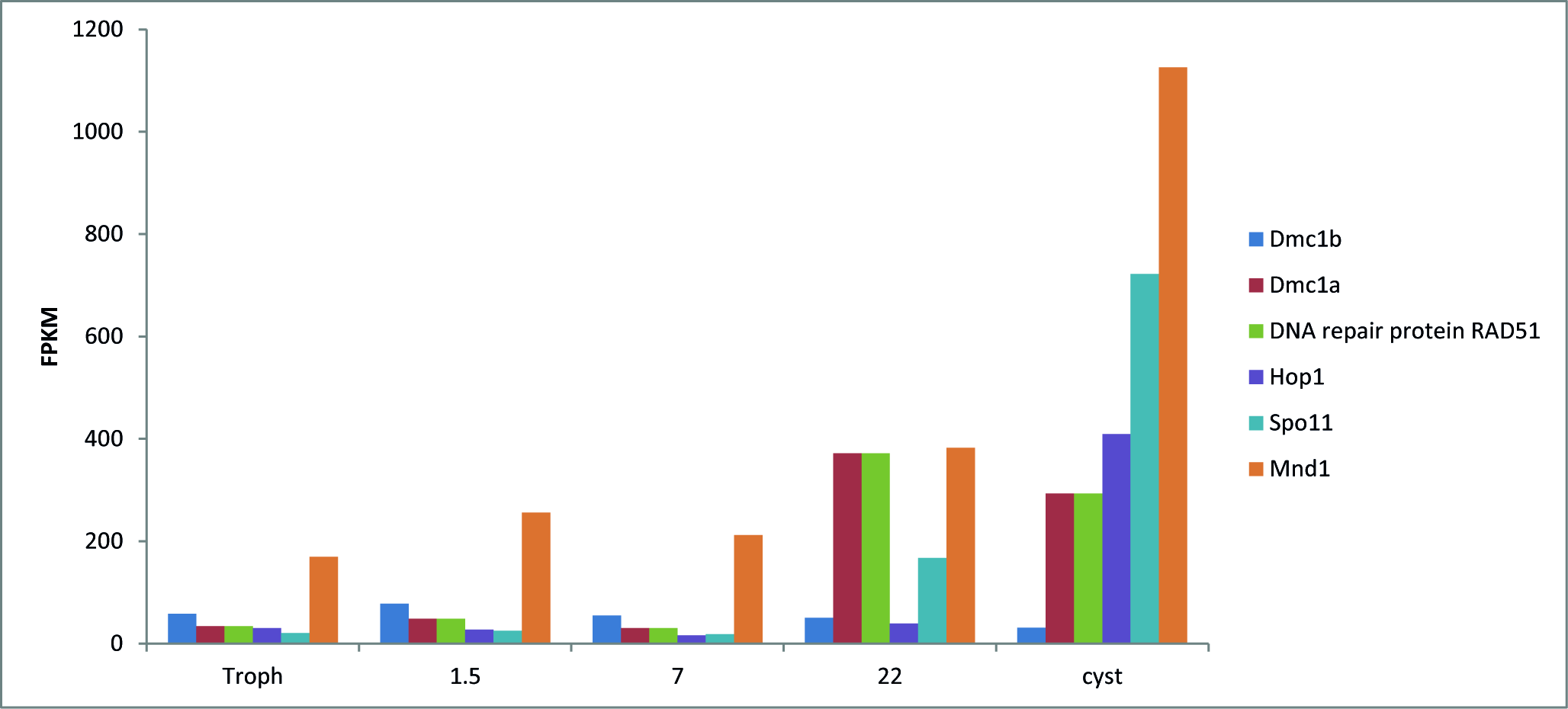

Supplement: S5 Fig — The meiosis related genes found in Giardia reveal differential expression during encystation with a marked up-regulation during later stages. (TIF) [file pntd.0004571.s010.tif]

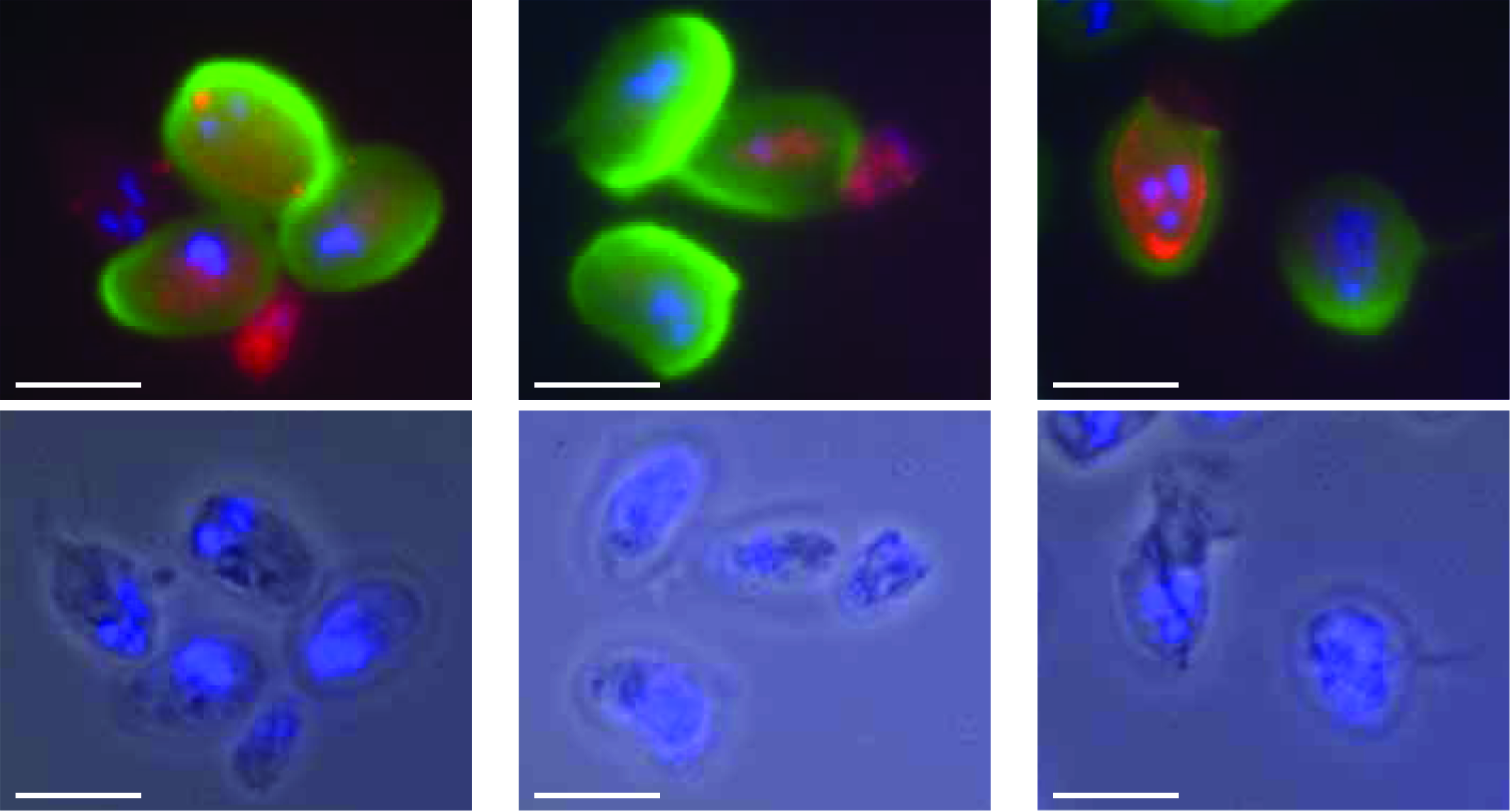

Supplement: S6 Fig — Water treated cysts expressing 23439-HA were excysted as described in Materials and Methods. Emerging excyzoites were fixed in solution and the HA-tagged protein was localized using an anti-HA antibody (red) in conjunction with an antibody to CWP1 (green) and DAPI for nuclear DNA staining. The tagged proteins appear on the surface of emerging excyzoites. Scale bars 10 µm. (TIF) [file pntd.0004571.s011.tif]

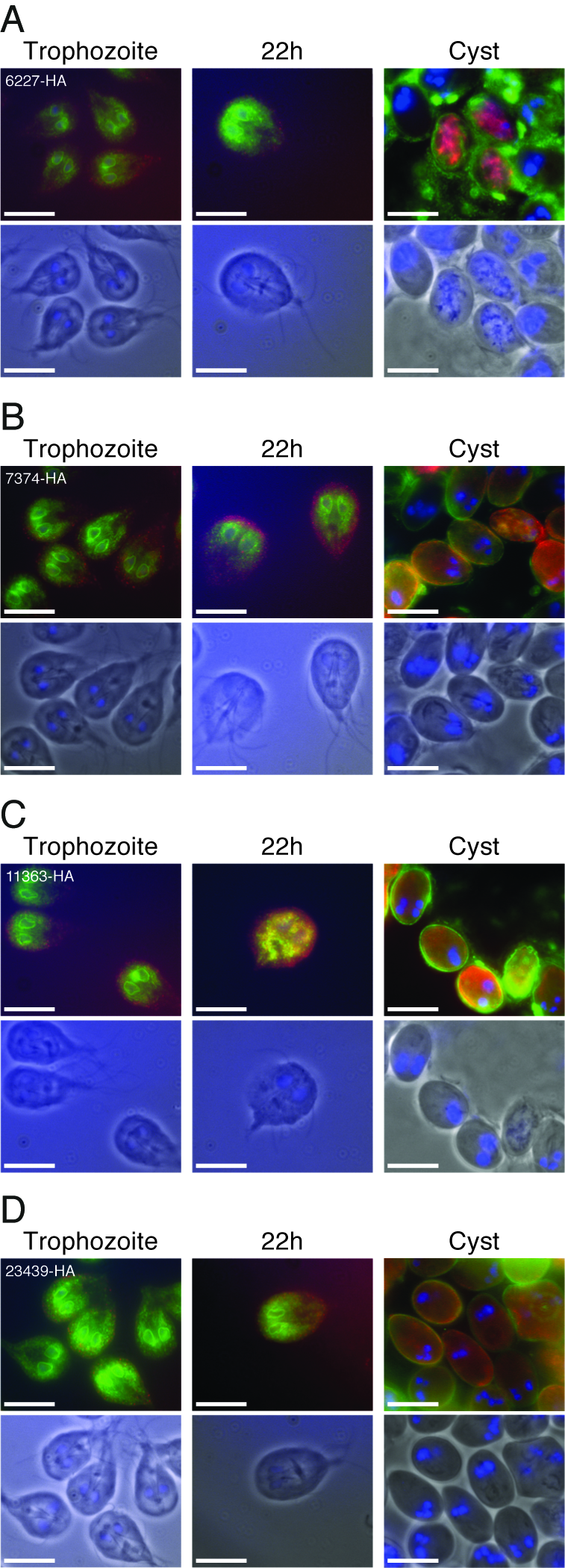

Supplement: S7 Fig — The proteins 6227, 7374, 11363 and 23439 display localizations in vesicle-like puncta and in order to investigate potential co-localization to ECVs, we used fluorescein labeled WGA. The HA-tagged strains of these proteins were subjected to encystation followed by immunofluorescence analysis to detect potential co-localizations. The WGA labeling (green) displayed ER-like staining and did not co-localize with any of the HA-tagged (red) proteins in encysting parasites or in cysts. Scale bars represent 10 μm. (TIF) [file pntd.0004571.s012.tif]
